# Traditional Chinese medicine for pediatric adenoid hypertrophy: an Umbrella review of methodological, reporting, and evidence quality

**DOI:** 10.3389/falgy.2026.1853582

**Published:** 2026-05-29

**Authors:** Xiaolan Shen, Guibin Deng, Sihan Chen

**Affiliations:** 1 The First College of Clinical Medical Sciences, China Three Gorges University, Yichang, Hubei, China; 2 Yichang Central People’s Hospital, Yichang, Hubei, China; 3 Yichang Huimin Hospital, Yichang, China

**Keywords:** adenoid hypertrophy, AMSTAR-2, GRADE system, PRISMA 2020, traditional Chinese medicine

## Abstract

**Objective:**

To conduct an umbrella review evaluating the methodological quality, reporting completeness, and certainty of evidence from systematic reviews and meta-analyses on traditional Chinese medicine (TCM) for pediatric adenoid hypertrophy (AH).

**Methods:**

A systematic search was conducted in PubMed, Web of Science, CNKI, VIP, and Wanfang databases from inception to March 2026, with no language restrictions. Eligible systematic reviews and meta-analyses of randomized controlled trials (RCTs) on TCM for pediatric AH were included. Two reviewers independently performed screening, data extraction, and quality assessment using AMSTAR-2, PRISMA 2020, and GRADE. Discrepancies were resolved by consulting a third reviewer.

**Results:**

Ten systematic reviews and meta-analyses encompassing 122 RCTs and 9,772 participants were included. All included reviews were rated as having low or critically low methodological quality and inconsistent reporting standards. Existing reviews reported possible improvements in clinical symptoms and adenoid-related outcomes with TCM interventions. However, these findings should be interpreted cautiously because of methodological limitations, risk of bias in the underlying trials, publication bias, imprecision, and clinical heterogeneity.

**Conclusion:**

Current review-level evidence on TCM interventions for pediatric AH remains methodologically weak and uncertain. The included systematic reviews showed low or critically low methodological quality, incomplete reporting, and limitations in the certainty of evidence. Future studies should prioritize well-designed randomized controlled trials, standardized outcome reporting, and rigorously conducted systematic reviews.

## 1 Introduction

Adenoid hypertrophy (AH) is a common upper airway disorder in children, characterized by nasal obstruction, mouth breathing, snoring, sleep disturbances, and recurrent otitis media (1–4). It primarily affects preschool and early school-aged children, exerting substantial adverse impacts on their growth, development, and quality of life (5–8). The pathogenesis of AH involves recurrent infections, allergic responses, and immune dysregulation, which collectively induce lymphoid hyperplasia and chronic inflammation of the adenoids (9–12). Current clinical interventions include glucocorticoid, antiallergic agents, antibiotics, and adenoidectomy. While surgical resection offers rapid symptom relief, it carries inherent risks of anesthesia, postoperative complications, and potential recurrence. Pharmacotherapy, on the other hand, is often constrained by prolonged treatment courses, poor patient adherence, and adverse effects (3, 13–15). Thus, exploring safe, effective non-surgical therapies suitable for pediatric populations has become an urgent clinical priority, and Traditional Chinese Medicine (TCM) has garnered growing attention in this context.

In recent years, TCM has been extensively utilized and investigated in the management of AH, with some studies reporting potential clinical benefits and favorable safety profiles (16, 17). Clinical investigations have employed diverse TCM modalities, including herbal decoctions, acupuncture, Tuina, and external therapies such as herbal patches or fumigation, either as monotherapy or in combination with Western medicine (18–21). Several systematic reviews and meta-analyses have synthesized these findings, indicating that TCM interventions can reduce adenoid volume, alleviate nasal obstruction, improve sleep quality, and modulate immune and inflammatory pathways (19, 22–25). Guided by the principles of holism and syndrome differentiation, TCM is hypothesized to exert multi-component, multi-target therapeutic effects (26, 27). Moreover, previous studies have suggested that TCM treatments may be relatively well tolerated in children. Nevertheless, TCM interventions have been increasingly investigated as non-surgical options for pediatric AH, the reliability of the existing evidence remains uncertain. Published systematic reviews vary in methodological rigor, intervention classification, outcome reporting, and assessment of bias, which limits confidence in broad claims regarding clinical effectiveness.

To address these limitations, our study aims to conduct a structured reappraisal of existing systematic reviews and meta-analyses on TCM for pediatric AH. Three internationally validated tools will be employed to ensure methodological rigor and transparency. The AMSTAR-2 instrument will assess methodological quality, with a focus on critical domains including study protocol registration, literature search comprehensiveness, and bias assessment (28). The PRISMA 2020 checklist will evaluate the completeness and clarity of reporting (29). And the GRADE approach will rate the certainty of evidence for key outcomes, including clinical efficacy, adenoid size reduction, and adverse events, by accounting for limitations such as bias risk, inconsistency, indirectness, imprecision, and publication bias (30–37).

However, existing systematic reviews have mainly focused on summarizing therapeutic effects, whereas the methodological robustness, reporting completeness, and certainty of evidence underlying these reviews remain unclear. An umbrella review is therefore needed to determine how reliable the current review-level evidence is and to identify methodological priorities for future trials and reviews.

## 2 Material and methods

### 2.1 Search strategy

This study comprehensively searched the PubMed, Web of Science, China Scientific Journal database (VIP) database, China National Knowledge Infrastructure (CNKI), and Wan Fang databases from inception to March 2026. There was no limitation in language, nation, publication as well as time. The search term are as follows:("adenoid hypertrophy"[Title/Abstract] OR "adenoidal hypertrophy"[Title/Abstract] OR "adenoid vegetation"[Title/Abstract] OR "adenoid hyperplasia"[Title/Abstract]) AND ("traditional Chinese medicine"[Title/Abstract] OR "Chinese herbal medicine"[Title/Abstract] OR decoction[Title/Abstract] OR acupuncture[Title/Abstract] OR tuina[Title/Abstract] OR massage[Title/Abstract] OR "Chinese patent medicine"[Title/Abstract] OR "integrated traditional Chinese and Western medicine"[Title/Abstract] OR "integrated Chinese and Western medicine"[Title/Abstract]) AND ("systematic review"[Title/Abstract] OR "systematic review"[Publication Type] OR "meta-analysis"[Title/Abstract] OR "Meta-Analysis"[Publication Type]). Search strategies in various databases are shown in Supplementary Tables S1, S2. The strategies were independently checked by two reviewers before final analysis.

### 2.2 Inclusion and exclusion criteria

The criteria of reviews are as following: (i) Research type: This study was designed as an umbrella review or an overview of systematic reviews, the included was systematic reviews and meta-analyses. (ii) Research subjects: Patients clinically diagnosed with AH, without concurrent infections, severe heart, lung or kidney function impairment. There are no restrictions on the age, gender, onset time, disease duration, race or nationality of the research subjects. (iii) Interventions: Patients in the treatment group were given traditional Chinese medicine decoctions, Chinese patent medicines, acupuncture, massage, external therapies of traditional Chinese medicine, combined traditional Chinese and Western medicine therapies, etc. Patients in the control group were given conventional treatment or placebo treatment. (iv) Main outcome measures: Clinical effective rate, Main Symptom Score, nasal obstruction scores, snoring scores, mouth breathing scores, A/N ratio. Secondary outcome measures include OSA-18 scores, Adverse effects, QSA-18, Sleep quality.

Because the purpose of this umbrella review was to appraise review-level evidence rather than to generate a new pooled estimate of treatment efficacy, different TCM modalities were included if they had been evaluated in eligible systematic reviews. Clinical heterogeneity across modalities was considered in the interpretation of findings and in the assessment of applicability.

Moreover, for each included review, the list of primary RCTs was extracted when available. Potential overlap of primary trials across reviews was examined and considered when interpreting the evidence.

The following articles will be excluded: (i) reviews not associated with AH or with other complications of AH. (ii) outside of systematic reviews or meta-analyses or network meta-analysis. (iii) reviews that are not treated with TCM. (iv) duplicate published reviews. (v) reviews with obvious flaws of data, outcomes and whole text.

### 2.3 Literature screening and data extraction

Before formal screening, the two reviewers (Xiaolan Shen and Guibin Deng) conducted pilot screening of a subset of records to calibrate the eligibility criteria. Any discrepancies were discussed and the criteria were refined before independent full screening and data extraction.

All retrieved records were imported into EndNote X7 for duplicate removal. Two reviewers independently screened the titles, abstracts, and full texts, extracted data, and performed quality assessments using the predefined evaluation tools. Disagreements were resolved through discussion. If consensus could not be reached, a third reviewer (Sihan Chen) was consulted. The information was extracted for each review, including journal, year of publication, first author, number of included RCTs and participants, intervention type, comparative, outcome indicators, and main conclusions.

### 2.4 Addition on overlap of primary RCTs

Revised English: To assess overlap of primary RCTs across the included reviews, we extracted the list of primary studies from each review when available and constructed a review-by-primary-study citation matrix. Duplicated RCTs were identified by comparing author names, publication year, journal, sample size, intervention/control descriptions, and outcome information. The corrected covered area (CCA) was calculated using the formula CCA = (N - r)/[r × (c - 1)], where N is the total number of primary-study occurrences across reviews, r is the number of unique primary studies, and c is the number of included reviews. Overlap was interpreted as slight (<5%), moderate (5% to <10%), high (10% to <15%), or very high (≥15%). The overlap results were considered when interpreting the certainty and applicability of the evidence.

### 2.5 Report quality and methodological evaluation

The methodological, reporting and evidence quality of all included systematic reviews or meta-analyses were respectively analyzed by using the AMSTAR-2 assessment, PRISMA 2020 system and GRADE tools (28, 29, 31, 38).

The AMSTAR-2 assessment is consisted of 16 items, with 7 critical items identified according to the guidance (items 2, 4, 7, 9, 11, 13, 15). Fully met the evaluation criteria punctuality, and evaluated as “Yes”. When the criteria are partially met, the evaluation is “ Partial Yes “. When no relevant information is reported in the system review “, the evaluation is “No”. At the end of the evaluation, they were divided into four diverse levels, which are described as high (one non-critical flaw), moderate (more than one non-critical flaw), low (one critical flaw, with or without non-critical flaws), and critically low (more than one critical flaw, with or without noncritical flaws) (32). The rationale of items of AMSTAR-2 is shown in Supplementary Table S3.

PRISMA 2020 statement is designed to assess the reporting completeness in reviews. This structure consists of seven aspects with the 42 items checklist, among which expand the checklist that specify the reporting recommendations and give an example for each item. The evaluation criteria consist of three parts respectively “Yes” (satisfies the criterion completely), “Partial Yes” (satisfies the criterion incompletely) or “No” (obviously does not satisfy the criterion), evaluation followed by assigning points of “1”, “0.5”, or “0”, with scores ranging from 0 to 42 (29). The PRISMA 2020 checklists are shown in Supplementary Table S4.

In this study, the GRADE tool was mainly used for assessing the quality of evidence in all included reviews. The result of evidence was classified as high (the true effect approach to the estimated effect), medium (the true value is likely to be close to the estimated effect value, but there is still the possibility that the two different substantial), low (the true value may be quite different from the estimate), and critically low (the true value may be quite different from the estimate). In addition, five aspects that could lead to the degradation of evidence quality were risk of bias, publication bias, imprecision, inconsistency, and indirect. All outcome measures were evaluated, with each downgrading factor rated as “not serious” or “serious” (leading to one level of downgrading, −1), and the included studies were upgraded to varying degrees based on effect size and the dose-response (leading to one level of upgrading, +1) (32, 34–37), Supplementary Table S5.

## 3 Results

### 3.1 Literature screening process and basic information

**Figure 1:**
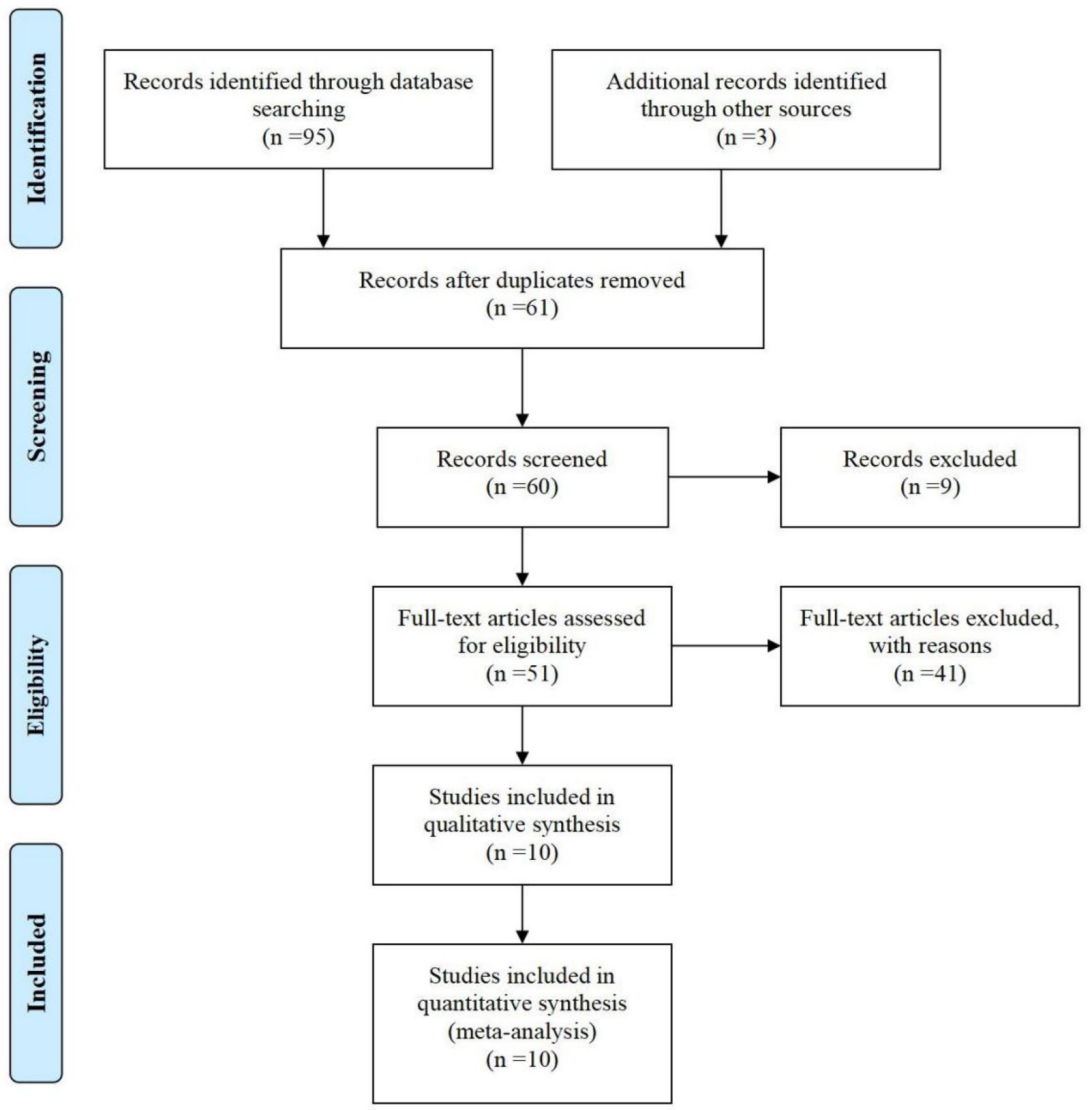
Flow diagram of the included study screening procedure.

A total of 98 studies related to TCM interventions for pediatric adenoidal hypertrophy were initially retrieved. After removing duplicates with EndNote software, 61 studies remained. Title and abstract screening excluded 9 studies that failed to met the specified inclusion criteria. Subsequent full-text assessment of the remaining 51 studies led to the inclusion of 10 systematic reviews and meta-analyses (19–25, 39–41), Figure 1.

The included studies were published from 2018 to 2024, and all assessed the methodological quality of the original studies using the Cochrane risk of bias tool. A total of 122 randomized controlled trials (RCTs) were identified, comprising 9,772 participants. The number of RCTs included in each systematic review ranged from 6 to 25, with corresponding sample sizes ranging from 353 to 2,008. Interventions mainly involved TCM tuina, single traditional Chinese medicine therapies, or integrated TCM and Western medicine, whereas controls included Western medicine, Chinese patent medicine, or conventional interventions excluding tuina. The main outcome measures were clinical effective rate, symptom scores, A/N ratio, adenoid volume, quality of life, and incidence of adverse events. The basic characteristics and conclusions are shown in Table 1.

**Table 1:** Basic information and characteristic of all included reviews.

Study	Years	RCT/Patients	Treatment intervention	Control intervention	Quality assessment	Conclusion
Guo	2022	10/624	massage	TCM or Western medicine	Cochrane	The incidence of adverse reactions in the treatment of children with adenoidal hypertrophy by massage is low, and it has a high safety. It can improve the clinical symptoms and signs, A/N values and quality of life of children with adenoidal hypertrophy. However, the overall evidence of efficacy is limited, and more multicenter, high-quality, large sample randomized controlled trials are needed to verify its efficacy.
Li	2023	6/353	massage	Except massage	Cochrane	Existing evidence indicated that massage alone or in combination with drugs is effective in the treatment of adenoid hypertrophy in children, and was significantly better than the control group. Nevertheless, due to the limitations of the number and quality of the samples, more high － quality ＲCTS are required to verify above conclusions
Song	2024	11/660	massage	Except massage	Cochrane	Pediatric tuina alone or in combination has better effectiveness and good safety on AH. However, the sample size of this study was small, most of the included studies had methodological deficiencies, and more high-quality evidence is needed to support the conclusions.
Zhu	2020	10/464	TCM + Western medicine	Western medicine	Cochrane	Based on the existing data and methods, the combined treatment of traditional Chinese medicine and Western medicine for adenoid hypertrophy in children has a reliable therapeutic effect. It is superior to the use of Western medicine alone in terms of treatment effectiveness and improvement of clinical symptoms, and there are no serious adverse reactions.
Cheng	2023	25/2008	TCM, or combination of TCM and Western medicine, or massage, or auricular cupuncture, or acupuncture.	Western medicine	Cochrane	Compared with Western medicine treatment, traditional Chinese medicine has more definite and reliable therapeutic effects for adenoid hypertrophy. When formulating prescriptions and selecting medications, it focuses on staged diagnosis and treatment, balancing both the symptoms and the root causes, and adopts flexible treatment methods.
Xu	2020	17/1339	TCM or TCM + Western medicine	Western medicine	Cochrane	The curative effect of traditional Chinese medicine in improving the main clinical symptoms of adenoid hypertrophy in children, reducing A/N ratio, reducing adenoid volume is significant. No serious adverse reactions are found. It is worthy of widely clinical application.
Ma	2020	14/1247	TCM	Western medicine	Cochrane	Traditional Chinese medicine for children with adenoidal hypertrophy can improve clinical effectiveness and improve the symptoms of nasal congestion, snoring, and mouth breathing, and reduce the adenoid volume with high safety. This conclusion still needs a large sample and high quality RCT to further verify.
Ren	2024	16/1206	TCM	Chinese patent medicine or Western medicine	Cochrane	In terms of reducing adenoid volume, improving clinical symptoms and quality of life, oral administration of traditional Chinese medicine is superior to conventional western medicine or Chinese patent medicine, and it is safe and worthy of promotion.
Sun	2019	13/1038	TCM	Western medicine	Cochrane	Chinese herbal medicine has good clinical efficacy and safety on pediatric adenoid hypertrophy, which need to be confirmed by high quality, multiple-centre, large sample randomized controlled trials.
Liu	2018	10/803	TCM	Western medicine	Cochrane	The use of Chinese medicine for the treatment of children with adenoid hypertrophy has good clinical efficacy.

### 3.2 Methodological quality assessment of included systematic reviews

The AMSTAR-2 scale was used to assess the methodological quality. The overall quality assessment revealed that none of the included studies were rated as high or moderate quality. There were 5 low quality studies (19, 20, 23, 24, 40) and 5 critically low quality studies (19, 21, 22, 25, 39). The scores of the individual studies ranged from 7.0 to 12.0, with the highest score (12.0) achieved by the Sun's study and the lowest (7.0) by the Liu's study.

**Figure 2:**
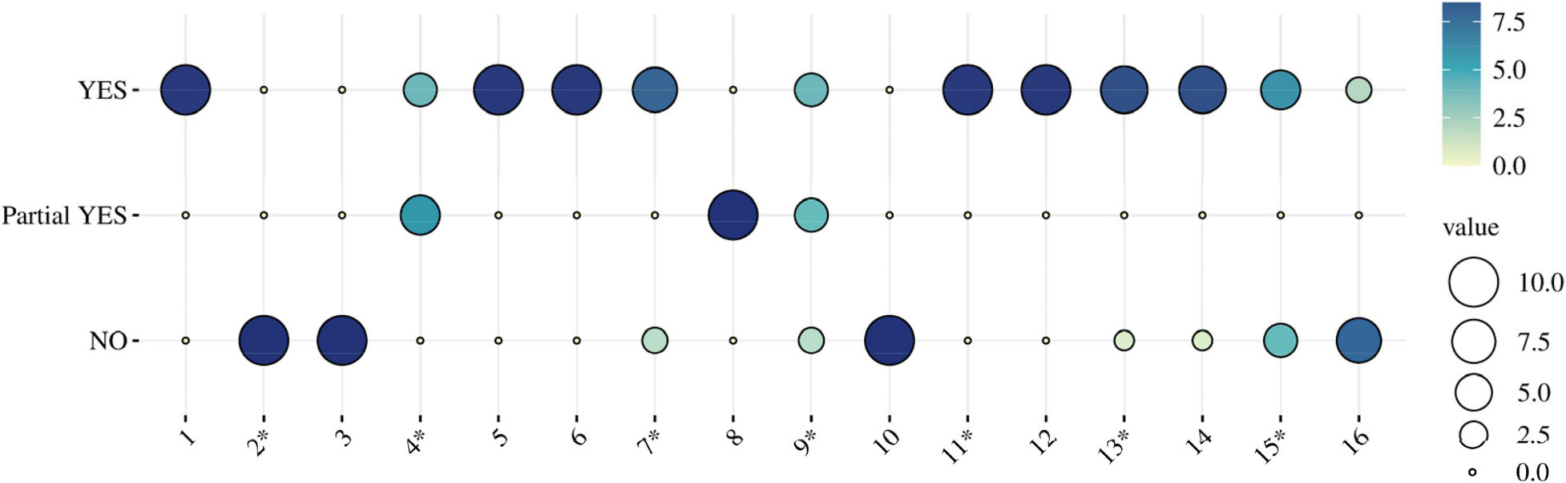
AMSTAR-2 score bubble chart.

The compliance with AMSTAR-2 items showed that all 10 studies fully adhered to item1, item5, item6, item11*, and item12, representing the most standardized methodological aspects. In contrast, item2* and item 10 were not reported in any study, identifying them as critical methodological gaps. Partial compliance was observed in item4* and item8, indicating ambiguities in defining participants and interventions and incomplete reporting of inclusion criteria, Figure 2. Key items showing deficiencies included item7* with 80.00% full compliance and 20.00% non-compliance, and item 9* with 40.00% full compliance, 40.00% partial compliance, and 20.00% non-compliance. Item13* and item14 showed 90.00% full compliance, while item15* had 60.00% full compliance. Item16 had only 20.00% full compliance. Regarding individual study performance, the Sun's study (12.0 points) ranked highest in methodological quality, failing only on item2* and item10 (24), while the Liu study (7.0 points) scored the lowest, with non-compliance in several key items such as item7*, item13*, and item14 (25). The detailed AMSTAR-2 scores and overall compliance are presented in Tables 2, 3.

**Table 2:** The evaluation results of the included reviews of AMASTAR-2.

Item	1	2*	3	4*	5	6	7*	8	9*	10	11*	12	13*	14	15*	16	Score	Quality
Guo	Y	N	N	PY	Y	Y	Y	PY	PY	N	Y	Y	Y	Y	N	N	9.5	Critically Low
Li	Y	N	N	PY	Y	Y	Y	PY	Y	N	Y	Y	Y	Y	Y	N	11	Low
Song	Y	N	N	PY	Y	Y	N	PY	PY	N	Y	Y	Y	Y	N	N	8.5	Critically Low
Zhu	Y	N	N	Y	Y	Y	Y	PY	Y	N	Y	Y	Y	Y	Y	N	11.5	Low
Cheng	Y	N	N	Y	Y	Y	Y	PY	PY	N	Y	Y	Y	Y	N	N	10	Critically Low
Xu	Y	N	N	PY	Y	Y	Y	PY	N	N	Y	Y	Y	Y	Y	N	10	Critically Low
Ma	Y	N	N	PY	Y	Y	Y	PY	Y	N	Y	Y	Y	Y	Y	N	11	Low
Ren	Y	N	N	Y	Y	Y	Y	PY	Y	N	Y	Y	Y	Y	Y	N	11.5	Low
Sun	Y	N	N	Y	Y	Y	Y	PY	PY	N	Y	Y	Y	Y	Y	Y	12	Low
Liu	Y	N	N	PY	Y	Y	N	PY	N	N	Y	Y	N	N	N	Y	7	Critically Low

Y:YES, N:NO, PY: Partial YES.

*Present key items.

**Table 3:** Methodological quality of all included reviews by AMSTAR-2 assessment.

Item	YES		Partial YES		NO	
	Frequency	Proportion(%)	Frequency	Proportion(%)	Frequency	Proportion(%)
1	10.00	100.00	0.00	0.00	0.00	0.00
2*	0.00	0.00	0.00	0.00	10.00	100.00
3	0.00	0.00	0.00	0.00	10.00	100.00
4*	4.00	40.00	6.00	60.00	0.00	0.00
5	10.00	100.00	0.00	0.00	0.00	0.00
6	10.00	100.00	0.00	0.00	0.00	0.00
7*	8.00	80.00	0.00	0.00	2.00	20.00
8	0.00	0.00	10.00	100.00	0.00	0.00
9*	4.00	40.00	4.00	40.00	2.00	20.00
10	0.00	0.00	0.00	0.00	10.00	10.00
11*	10.00	100.00	0.00	0.00	0.00	0.00
12	10.00	100.00	0.00	0.00	0.00	0.00
13*	9.00	90.00	0.00	0.00	1.00	10.00
14	9.00	90.00	0.00	0.00	1.00	10.00
15*	6.00	60.00	0.00	0.00	4.00	40.00
16	2.00	20.00	0.00	0.00	8.00	80.00

Y:YES, N:NO, PY: Partial YES.

*Present key items.

A central finding of this umbrella review was that none of the included reviews achieved high or moderate methodological quality according to AMSTAR-2. All were rated as low or critically low. This substantially limits the certainty of conclusions regarding the effectiveness of TCM interventions for pediatric AH.

### 3.3 Reporting completeness assessment of included systematic reviews

The PRISMA 2020 statement was used to evaluate reporting completeness, with a maximum score of 42, with criteria for full compliance (Y), partial compliance (PY), and non-compliance (N). The reporting scores of the studies ranged from 19.5 to 34.5, with the Sun study scoring the highest (34.5) and the Liu study the lowest (19.5), indicating a 15-point gap and significant variation in reporting completeness.

**Figure 3:**
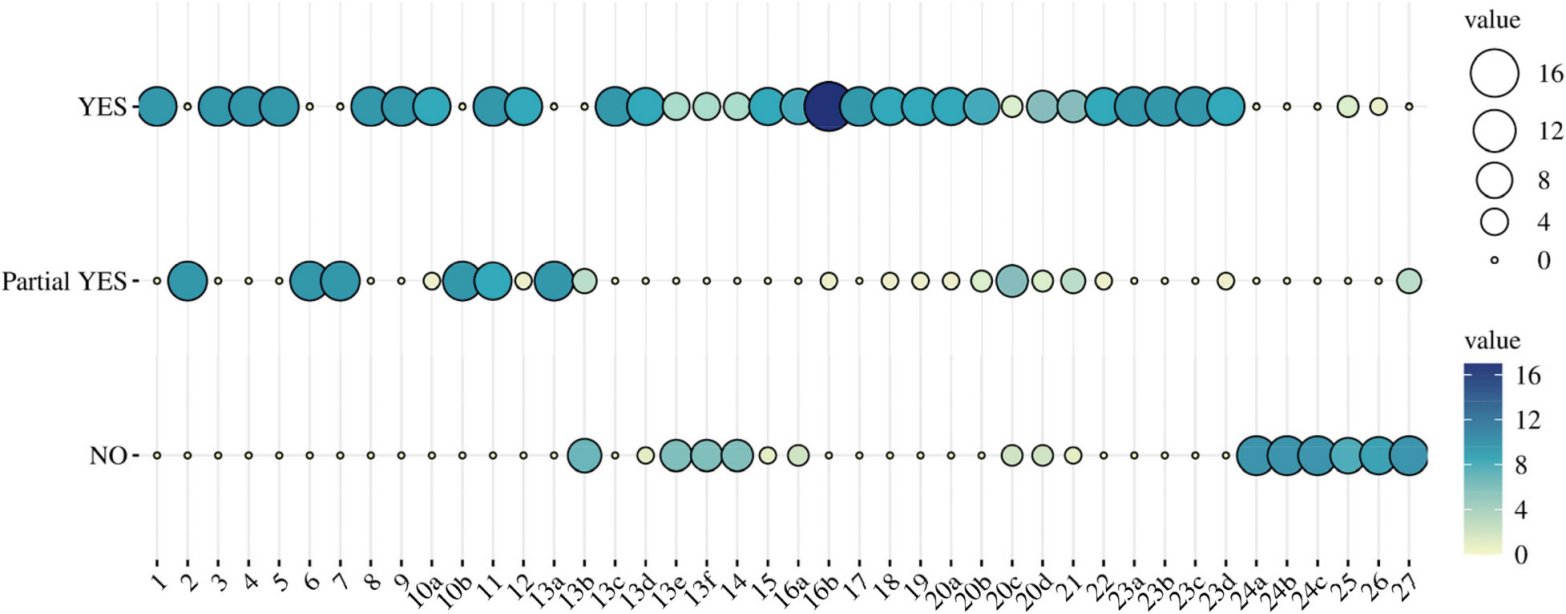
PRISMA 2020 score bubble chart.

In terms of reporting characteristics, the Title (item 1), introduction (items 3–5), core results items (item 17), and basic discussion items (items 23a–23c) were fully reported in all studies, representing the most standardized aspects. However, the abstract (item 2), search strategy details in the Methods (item 6), data extraction tools (item 7), and study selection process (item 13a) were only partially complied with by all studies, pointing to incomplete abstract information and insufficient methodological disclosure, Figure 3. Notably, no study reported the analysis of risk of bias impact (items 24a–24c), and 90% of studies failed to identify knowledge gaps or future research directions (item 27). In the Methods module, items 13b, 13e, 13f, and 14 showed poor compliance (≤ 40%), mainly due to incomplete reporting of study selection, risk of bias assessment, and data synthesis methods. The results (items 20c, 20d) and discussion modules (items 25, 26) also exhibited low compliance, with study limitations (item 25) and evidence acquirement (item 26) showing full compliance rates of only 20% and 10%, respectively. The Sun study (34.5 points) showed >90% compliance in the methods, results, and discussion modules, with only a few partially complied items, while the Liu study (19.5 points) had over 50% non-compliance, especially in methodological details and evidence interpretation. Detailed scores and compliance are shown in Tables 4, 5.

**Table 4:** The evaluation results of the included reviews of PRISMA 2020.

Item	Guo	Li	Song	Zhu	Cheng	Xu	Ma	Ren	Sun	Liu
1	Y	Y	Y	Y	Y	Y	Y	Y	Y	Y
2	PY	PY	PY	PY	PY	PY	PY	PY	PY	PY
3	Y	Y	Y	Y	Y	Y	Y	Y	Y	Y
4	Y	Y	Y	Y	Y	Y	Y	Y	Y	Y
5	Y	Y	Y	Y	Y	Y	Y	Y	Y	Y
6	PY	PY	PY	PY	PY	PY	PY	PY	PY	PY
7	PY	PY	PY	PY	PY	PY	PY	PY	PY	PY
8	Y	Y	Y	Y	Y	Y	Y	Y	Y	Y
9	Y	Y	Y	Y	Y	Y	Y	Y	Y	Y
10a	Y	Y	Y	Y	Y	Y	Y	Y	Y	PY
10b	PY	PY	PY	PY	PY	PY	PY	PY	PY	PY
11	Y	Y	Y	Y	Y	Y	Y	Y	Y	Y
12	Y	Y	Y	Y	Y	Y	Y	Y	Y	PY
13a	PY	PY	PY	PY	PY	PY	PY	PY	PY	PY
13b	PY	N	N	PY	N	N	N	PY	N	N
13c	Y	Y	Y	Y	Y	Y	Y	Y	Y	Y
13d	Y	Y	Y	Y	Y	Y	Y	Y	Y	N
13e	N	N	Y	Y	N	N	N	Y	Y	N
13f	N	Y	N	Y	N	N	N	Y	Y	N
14	N	Y	N	Y	N	N	Y	N	Y	N
15	Y	Y	Y	Y	Y	Y	Y	Y	Y	N
16a	Y	Y	N	Y	Y	Y	Y	Y	Y	N
16b	Y	Y	N	Y	Y	Y	Y	Y	Y	N
17	Y	Y	Y	Y	Y	Y	Y	Y	Y	Y
18	Y	Y	Y	Y	Y	Y	Y	Y	Y	PY
19	Y	Y	Y	Y	Y	Y	Y	Y	Y	PY
20a	Y	Y	Y	Y	Y	Y	Y	Y	Y	PY
20b	Y	Y	Y	Y	Y	Y	PY	Y	Y	PY
20c	PY	PY	PY	Y	PY	PY	PY	N	Y	N
20d	Y	Y	PY	Y	PY	Y	Y	N	Y	N
21	PY	PY	PY	Y	Y	Y	Y	Y	Y	N
22	Y	Y	Y	Y	Y	Y	Y	Y	Y	PY
23a	Y	Y	Y	Y	Y	Y	Y	Y	Y	Y
23b	Y	Y	Y	Y	Y	Y	Y	Y	Y	Y
23c	Y	Y	Y	Y	Y	Y	Y	Y	Y	Y
23d	Y	Y	Y	Y	Y	Y	Y	Y	Y	PY
24a	N	N	N	N	N	N	N	N	N	N
24b	N	N	N	N	N	N	N	N	N	N
24c	N	N	N	N	N	N	N	N	N	N
25	N	N	N	N	N	N	N	N	Y	Y
26	N	N	N	N	N	N	N	N	Y	N
27	N	N	N	N	N	N	N	N	N	N
Points	29	30.5	27	33	28.5	29	29.5	30	34.5	19.5

Y:YES, N:NO, PY: Partial YES.

**Table 5:** Reporting completeness of all included reviews by PRISMA 2020 system.

Section	Item	YES		Partial YES		NO	
		Frequency	proportion(%)	Frequency	proportion(%)	Frequency	proportion(%)
Title	1	10.00	100.00	0.00	0.00	0.00	0.00
Abstract	2	0.00	0.00	10.00	100.00	0.00	0.00
Introduction	3	10.00	100.00	0.00	0.00	0.00	0.00
	4	10.00	100.00	0.00	0.00	0.00	0.00
Methods	5	10.00	100.00	0.00	0.00	0.00	0.00
	6	0.00	0.00	10.00	100.00	0.00	0.00
	7	0.00	0.00	10.00	100.00	0.00	0.00
	8	10.00	100.00	0.00	0.00	0.00	0.00
	9	10.00	100.00	0.00	0.00	0.00	0.00
	10a	9.00	90.00	1.00	10.00	0.00	0.00
	10b	0.00	0.00	10.00	100.00	0.00	0.00
	11	10.00	100.00	9.00	90.00	0.00	0.00
	12	9.00	90.00	1.00	10.00	0.00	0.00
	13a	0.00	0.00	10.00	100.00	0.00	0.00
	13b	0.00	0.00	3.00	30.00	7.00	70.00
	13c	10.00	100.00	0.00	0.00	0.00	0.00
	13d	9.00	90.00	0.00	0.00	1.00	10.00
	13e	4.00	40.00	0.00	0.00	6.00	60.00
	13f	4.00	40.00	0.00	0.00	6.00	60.00
	14	4.00	40.00	0.00	0.00	6.00	60.00
	15	9.00	90.00	0.00	0.00	1.00	10.00
Results	16a	8.00	80.00	0.00	0.00	2.00	20.00
	16b	17.00	170.00	1.00	10.00	0.00	0.00
	17	10.00	100.00	0.00	0.00	0.00	0.00
	18	9.00	90.00	1.00	10.00	0.00	0.00
	19	9.00	90.00	1.00	10.00	0.00	0.00
	20a	9.00	90.00	1.00	10.00	0.00	0.00
	20b	8.00	80.00	2.00	20.00	0.00	0.00
	20c	2.00	20.00	6.00	60.00	2.00	20.00
	20d	6.00	60.00	2.00	20.00	2.00	20.00
	21	6.00	60.00	3.00	30.00	1.00	10.00
	22	9.00	90.00	1.00	10.00	0.00	0.00
Discussion	23a	10.00	100.00	0.00	0.00	0.00	0.00
	23b	10.00	100.00	0.00	0.00	0.00	0.00
	23c	10.00	100.00	0.00	0.00	0.00	0.00
	23d	9.00	90.00	1.00	10.00	0.00	0.00
Other information	24a	0.00	0.00	0.00	0.00	10.00	100.00
	24b	0.00	0.00	0.00	0.00	10.00	100.00
	24c	0.00	0.00	0.00	0.00	10.00	100.00
	25	2.00	20.00	0.00	0.00	8.00	80.00
	26	1.00	10.00	0.00	0.00	9.00	90.00
	27	0.00	0.00	3.00	30.00	10.00	100.00

### 3.4 Evidence quality assessment of included systematic reviews

The GRADE system was used to assess the quality of evidence for 43 core outcome indicators across 10 studies.Based on five downgrading factors and upgrading factors, the evidence quality was classified into four grades: high, moderate, low, and critically low. Overall, no high-quality evidence was identified. Of the 43 indicators, 25 were rated as moderate-quality (58.14%), 7 as low-quality (16.28%), and 11 as critically low-quality (25.58%). This distribution shows that moderate-quality evidence predominated, with nearly half of the evidence categorized as low or critically low.

Analysis of evidence quality characteristics for specific outcome indicators revealed that key measures such as nasal obstruction score, snoring score, mouth breathing score, adenoid volume, adverse event incidence, and recurrence rate had more than 80% moderate-quality evidence, as these original studies exhibited low risk of bias and consistent data. In contrast, indicators such as quality of life scores (QSA-18/OSA-18), sleep quality, and main symptom scores showed over 70% critically low-quality evidence. The main downgrading factors for these included lack of blinding in the original studies, imprecise results, and publication bias. The clinical effective rate varied across studies: moderate-quality in 5 studies (21, 22, 24, 40, 41), low-quality in the Song and Liu studies (19, 25), and critically low-quality in the Zhu and Ma studies (20, 23), reflecting differences in sample size and risk of bias control.

Further analysis of downgrading factors revealed that all outcome indicators were downgraded due to risk of bias (−1), as most original RCTs had unclear randomization methods, inadequate allocation concealment, and lack of blinding. Additional downgrading factors included publication bias (−1) and imprecision (−1), with some indicators further downgraded due to inconsistency (−1), such as sleep quality (*I*^2^ = 98%) and A/N ratio (*I*^2^ = 95.6%). No outcome indicators were downgraded due to indirectness, and no upgrading factors were identified across all studies, Table 6.

**Table 6:** The evaluation results of the included reviews of GRADE system.

Study	Years	RCT/Patients	Outcomes	Effect index	95%CI	*I*^2^(%)	Risk of bias	inconsistency	indirectness	imprecision	Publication bias	Rating up	Quality
Guo	2022	10/624	Adverse effects	RR = 0.26	0.07,0.99	0	−1	−1	0	−1	−1	1	Critically low
			Clinical effective rate	RR = 1.30	1.19,1.41	0	−1	0	0	0	−1	0	Low
			Main Symptom Score	MD = −0.75	−2.24,0.74	0	−1	−1	0	−1	−1	0	Critically low
			A/N ratio	RR = 0.01	−0.01,0.03	0	−1	0	0	0	−1	0	Low
			QSA-18	RR = -8.62	-10.78,-6.46	0	-1	-1	0	-1	-1	0	Critically low
			Sleep quality	RR = -0.84	-3.92,2.23	98	-1	-1	0	-1	-1	0	Critically low
Li	2023	6/353	Clinical effective rate	RR = 1.24	1.13,1.35	7	-1	0	0	0	0	0	Medium
			Main Symptom Score	RR = 1.28	1.11,1.48	0	-1	0	0	0	0	0	Medium
			A/N ratio	RR = -0.36	0. 87,0. 15	86	-1	0	0	0	0	0	Medium
			Quality of Life Score	RR = -8.92	-11.11,-6.73	0	-1	-1	0	-1	0	0	Critically low
Song	2024	11/660	Clinical effective rate	RR = 1.22	1.13,1.30	7	-1	0	0	0	-1	0	Low
			Main Symptom Score	RR = -4.66	-6.66,-2.44	89	-1	-1	0	-1	-1	0	Critically low
Zhu	2020	10/464	Clinical effective rate	OR = 4.27	2.50,7.31	0	-1	-1	0	-1	0	0	Critically low
			nasal obstruction scores	MD = -0.64	-0.94,-0.35	86	-1	0	0	0	0	0	Medium
			Snoring scores	MD = -0.66	-1.11,-0.21	94	-1	0	0	0	0	0	Medium
			A/N ratio	MD = -1.12	-1.61,-0.63	86	-1	0	0	0	0	0	Medium
Cheng	2023	25/2008	Clinical effective rate	OR = 4.14	3.16,5.42	0	-1	0	0	0	0	0	Medium
			nasal obstruction scores	MD = -1.17	-1.74,-0.60	94.4	-1	0	0	0	0	0	Medium
			Snoring scores	MD = -1.23	-1.73,-0.74	92.8	-1	0	0	0	0	0	Medium
			mouth breathing scores	MD = -1.46	-2.09,-0.82	95.3	-1	-1	0	-1	0	0	Critically low
			A/N ratio	MD = -1.08	-1.47,-0.69	86.7	-1	0	0	0	0	0	Medium
			OSA-18 scores	MD = -1.80	-2.92,-0.68	95.8	-1	-1	0	-1	0	0	Critically low
			Recurrence rate	OR = 0.24	0.12,0.49	0	-1	0	0	0	0	0	Medium
Xu	2020	17/1339	Clinical effective rate	RR = 1.33	1.25,1.41	60	-1	0	0	0	0	0	Medium
			nasal obstruction scores	WMD=-0.61	-0.82,-0.39	74.9	-1	0	0	0	0	0	Medium
			Snoring scores	WMD=-0.82	-1.14,-0.5	86.3	-1	0	0	0	0	0	Medium
			mouth breathing scores	WMD=-0.54	-0.72,-0.36	68.6	-1	0	0	0	0	0	Medium
			A/N ratio	WMD=-0.11	-0.16,-0.55	95.6	-1	0	0	0	0	0	Medium
			Adenoid size	WMD=-0.63	-0.78,-0.48	33.1	-1	0	0	0	0	0	Medium
Ma	2020	14/1247	Clinical effective rate	OR = 3.79	2.73,5.25	0	-1	-1	0	-1	0	0	Critically low
			nasal obstruction scores	MD = -0.61	-0.70,-0.51	0	-1	0	0	0	0	0	Medium
			Snoring scores	MD = -0.55	-0.67,-0.42	46	-1	0	0	0	0	0	Medium
			mouth breathing scores	MD = -0.47	-0.57,-0.37	0	-1	0	0	0	0	0	Medium
			A/N ratio	MD = -1.28	-1.95,-0.61	90	-1	-1	0	-1	0	0	Critically low
			Adenoid size	MD = -0.67	-0.83,-0.50	0	-1	0	0	0	0	0	Medium
			Adverse effects	MD = 0.26	0.12,0.57	42	-1	0	0	0	0	0	Medium
Ren	2024	16/1206	Clinical effective rate	RR = 1.36	1.19,1.55	0	-1	0	0	0	0	0	Medium
			nasal obstruction scores	MD = -0.59	-0.68,-0.50	2	-1	0	0	0	0	0	Medium
			Snoring scores	MD = -0.53	-0.61,-0.45	29	-1	0	0	0	0	0	Medium
			mouth breathing scores	MD = -0.45	-0.60,-0.29	0	-1	0	0	0	0	0	Medium
			A/N ratio	MD = -0.12	-0.17,-0.08	95	-1	0	0	0	0	0	Medium
			Adenoid size	MD = -0.67	-0.84,-0.51	31	-1	0	0	0	0	0	Medium
			OSA-18 scores	MD = -7.69	-9.69,-5.69	0	-1	-1	0	-1	0	0	Critically low
			Safety	RR = 0,36	0.20,0.66	19	-1	0	0	0	0	0	Medium
Sun	2019	13/1038	Clinical effective rate	RR = 1.33	1.24,1.43	25	-1	0	0	0	0	0	Medium
			A/N ratio	MD = -0.04	-0.05,-0.03	0	-1	0	0	0	0	0	Medium
			OSA-18 scores	MD = -4.77	-8.35,-1.20	68	-1	-1	0	-1	0	0	Critically low
			nasal obstruction scores	MD = -0.56	-0.68,-0.45	38	-1	0	0	0	0	0	Medium
			Snoring scores	MD = -0.46	-0.62,-0.30	0	-1	0	0	0	0	0	Medium
			mouth breathing scores	MD = -0.52	-0.66,-0.39	0	-1	0	0	0	0	0	Medium
Liu	2018	10/803	Clinical effective rate	OR = 2.06	1.45, 2.96	Not Mentioned	-1	0	0	0	-1	0	Low
			Recurrence rate	OR = 3.05	2.11, 4.56	Not Mentioned	-1	0	0	0	-1	0	Low

Although several outcomes were rated as moderate certainty by GRADE, these ratings should not be interpreted as definitive evidence of clinical effectiveness, given the low or critically low methodological quality of the included reviews and the risk of bias in the underlying trials.

### 3.5 Comprehensive quality evaluation of included systematic reviews

**Figure 4:**
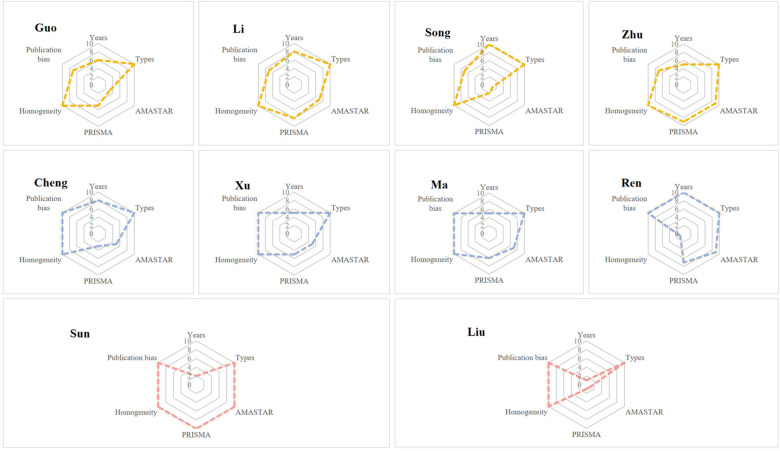
Radar chart for system evaluation.

A comprehensive quality score was calculated by integrating the results of AMSTAR-2, PRISMA 2020, homogeneity, and publication bias assessments. The comprehensive scores of individual studies ranged from 5.50 to 8.67. The Sun's study (8.67 points) achieved the highest scores in both AMSTAR-2 and PRISMA 2020 (24), with high homogeneity (*I*^2^ < 40%) and publication bias assessed using funnel plots, demonstrating strong performance across all dimensions. The Li and Zhu studies (both 8.33 points) exhibited moderate to high methodological and reporting completeness (20, 40), with deficiencies primarily in the absence of grey literature searches and the impact of risk of bias on results. The Liu's study (5.50 points) received the lowest scores in both AMSTAR-2 and PRISMA 2020 (25), with numerous methodological weaknesses and incomplete reporting, despite the assessment of publication bias using funnel plots. Additionally, 90% of the studies demonstrated high homogeneity (*I*^2^ < 50%), and 70% assessed publication bias using funnel plots. However, non-standard methodologies and incomplete reporting were common limitations across all included studies, Figure 4.

### 3.6 Overlap of primary RCTs across included reviews

Revised English: A citation matrix was constructed to assess overlap of primary RCTs across the included reviews. The 10 reviews contained 132 primary-study occurrences in total. After duplicate removal, 122 unique RCTs remained, indicating 10 repeated trial occurrences across reviews. The calculated CCA was 0.91%: CCA=(132–122)/[122 × (10-1)]×quantitative overlap was low, repeated inclusion of individual RCTs may still contribute to overconfidence in apparently consistent findings, particularly when the same trials have unclear risk of bias or report positive results. Therefore, overlap was considered when interpreting the certainty and applicability of the current review-level evidence.

## 4 Discussion

### 4.1 Methodological quality deficiencies and implications

Our study demonstrates significant strengths in methodological evaluation. Firstly, the AMSTAR-2 tool was used for a comprehensive assessment of the quality of the included systematic reviews, ensuring transparency and a systematic approach (42–44). By examining 16 evaluation items, the study not only identified key methodological deficiencies in existing research but also provided clear recommendations for future improvement. Specifically, the study emphasizes the importance of protocol registration and the use of the PICO framework to enhance transparency in study design (45). This focus on minimizing selective outcome reporting and post protocol adjustments can improve the reliability and consistency of study findings.

Furthermore, we addresses the impact of missing grey literature. Despite 20% of the studies failing to search for grey literature or report its results, this omission likely contributed to publication bias and overestimation of the clinical efficacy of TCM interventions. The study stresses the importance of including grey literature in future research to avoid bias and improve the comprehensiveness of findings. While many of the included studies had methodological shortcomings, they also exhibited strengths, particularly in research design and data analysis. Some studies used RCT designs, providing reliable clinical data supporting the efficacy of TCM treatments for pediatric adenoidal hypertrophy (46). Although certain studies had reporting deficiencies, their statistical rigor still provided a solid foundation for future systematic reviews and meta-analyses. Our study emphasizes the lack of comprehensive bias risk analysis as a significant shortcoming in current research and offers guidance for future studies on how to perform thorough bias assessments. Additionally, 40% of the studies did not disclose conflicts of interest, and 80% did not report funding sources, further highlighting the need for improved transparency regarding financial influences.

Overall, the central finding of this umbrella review is that the current review-level evidence on TCM interventions for pediatric AH remains methodologically weak and uncertain. These limitations reduce confidence in the conclusions of the included reviews, even when statistically significant effects were reported. Therefore, the findings of this umbrella review should be understood primarily as an appraisal of evidence limitations rather than as confirmation of therapeutic efficacy.

### 4.2 Reporting completeness defects and causes

The PRISMA 2020 evaluation revealed significant gaps in reporting completeness, with an average score of 29.2. The PRISMA 2020 assessment showed considerable variability in reporting completeness. Although some sections such as the title, introduction, and selected core results were generally reported, important methodological details were often incomplete, including full search strategies, data extraction procedures, study selection details, risk-of-bias impact, certainty assessment, limitations, and implications for future research. However, the included studies also demonstrate notable strengths. Most studies excelled in reporting key sections, including the title, abstract, introduction, and methods, with a 100% compliance rate, reflecting the research teams' commitment to transparency and adherence to reporting standards.

In the methods section, while some studies had minor deficiencies in detail, most reported study selection criteria and intervention descriptions with high compliance. Several studies also provided detailed result analyses and performed subgroup analyses for specific clinical populations, enhancing the clinical relevance of their findings. Although some studies lacked comprehensive reporting on bias risk and study limitations, they still offered valuable data for guiding future research.

Despite shortcomings in result analysis and evidence interpretation, several studies provided high-quality data synthesis and conducted heterogeneity and subgroup analyses. These strengths contribute to the clinical utility of the studies and provide a solid foundation for future systematic reviews and meta-analyses.

### 4.3 Evidence quality characteristics and downgrading factors

The GRADE evaluation revealed that the overall quality of evidence in the included studies was predominantly low or critically low, with nearly half of the evidence rated as low or critically low. The low certainty of evidence may be explained by weaknesses in the original trials, including unclear random-sequence generation, inadequate allocation concealment, lack of blinding, small sample sizes, selective outcome reporting, and insufficient follow-up. In addition, the predominance of positive findings in published reviews raises concerns about publication bias. However, the included studies demonstrated several notable strengths. Most studies adopted strict randomized controlled trial designs, indicating potential benefits of traditional Chinese medicine in treating children's adenoid hypertrophy. In particular, objective outcome indicators such as clinical therapeutic efficiency and main symptom scores consistently showed positive effects. Moreover, some studies conducted heterogeneity and subgroup analyses, providing certain guidance for the clinical relevance of their research results. For example, Cheng's study performed subgroup analyses, verifying the efficacy of TCM treatments across different clinical populations, thus providing valuable evidence for clinical application (21).

Despite the methodological shortcomings, particularly in terms of bias risk and publication bias, most of the included studies remain reasonably reliable. These studies demonstrated high compliance in reporting key sections such as the title, abstract, and methods, ensuring transparency and repeatability. However, the main sources of bias stemmed from unclear randomization and the lack of blinding, while some studies failed to adequately report study limitations and bias risk, which impacted the interpretation and applicability of the results.

Future studies need to focus on addressing issues related to blinding, sample size, and publication bias to further enhance the quality of evidence. The use of objective outcome measures and third-party assessments can help reduce bias risk, thereby improving the reliability of results. Moreover, increasing grey literature inclusion and conducting quantitative publication bias analysis will contribute to improving the comprehensiveness of systematic reviews, reducing the impact of publication bias, and providing more reliable evidence for clinical decision-making.

### 4.4 Comprehensive quality evaluation and clinical application value

Comprehensive quality analysis indicated that the included studies exhibited good homogeneity and a high rate of publication bias assessment, but common shortcomings included non-standard methodologies and incomplete reporting, resulting in a mean comprehensive quality score of only 7.58. This limits the clinical applicability of the findings. From a referable perspective, TCM interventions for pediatric adenoidal hypertrophy showed potential benefits in improving symptoms such as nasal obstruction, snoring, and mouth breathing, reducing the A/N ratio and adenoid volume, and decreasing the incidence of adverse reactions. The quality of evidence for these outcomes is moderate, making it a suitable reference for clinical non-surgical treatments. However, from a cautious application perspective, some evidence related to quality of life, sleep quality, and clinical effective rate was rated as low or critically low quality, with poor consistency across studies. Clinical application should be tailored to the specific conditions of the child, as these interventions cannot be blindly promoted without further high-quality studies for verification. Given that all included reviews were rated as low or critically low in methodological quality, the findings should be interpreted primarily as evidence of possible benefit.

Clinical heterogeneity is an important limitation of the current evidence base. The included reviews covered heterogeneous TCM modalities, including herbal medicine, Chinese patent medicine, acupuncture, Tuina or massage, external therapies, and combined TCM-Western interventions. These modalities differ substantially in theoretical basis, treatment procedures, practitioner dependence, treatment duration, and comparative interventions. Outcome definitions and measurement tools also varied across reviews. Therefore, broad conclusions about TCM should not be directly applied to any single modality. Future reviews should classify interventions more precisely, conduct modality-specific analyses when sufficient data are available, and avoid pooling clinically distinct interventions without adequate justification.

However, we also should consider clinical heterogeneity when interpreting the findings. The included reviews evaluated diverse TCM modalities and combined treatment strategies, and outcome definitions varied across studies. Therefore, broad conclusions across all TCM interventions may not be directly applicable to any single modality.

## 5 Conclusions

Current systematic reviews suggest possible benefits of TCM interventions for pediatric adenoid hypertrophy, but the review-level evidence remains methodologically weak and uncertain. Because all included reviews were rated as low or critically low quality and several outcomes were limited by risk of bias, publication bias, imprecision, overlap, and clinical heterogeneity, no firm clinical recommendation can be made based on the current evidence. Further well-designed randomized trials and rigorously conducted systematic reviews are needed to clarify the effectiveness and safety of specific TCM modalities for pediatric AH.

## References

[1] Niedzielski A Chmielik LP Mielnik-Niedzielska G Kasprzyk A Bogusławska J. Adenoid hypertrophy in children: a narrative review of pathogenesis and clinical relevance. BMJ Paediatr Open. (2023) 7(1):pp. 10.1136/bmjpo-2022-001710 PMC10106074 37045541

[2] Ma Y Xie L Wu W. The effects of adenoid hypertrophy and oral breathing on maxillofacial development: a review of the literature. J Clin Pediatr Dent. (2024) 48(1):1–6. 10.22514/jocpd.2024.001 38239150

[3] Zhu Y Wei P Kou W Yao H. [Strategies for preventing postoperative recurrence of adenoid hypertrophy]. Lin Chuang Er Bi Yan Hou Tou Jing Wai Ke Za Zhi. (2022) 36(10):807–12. 10.13201/j.issn.2096-7993.2022.10.016 36217664 PMC10128558

[4] Zhang J Fu Y Wang L Wu G. Adenoid facies: a long-term vicious cycle of mouth breathing, adenoid hypertrophy, and atypical craniofacial development. Front Public Health. (2024) 12:1494517. 10.3389/fpubh.2024.1494517 39726660 PMC11669592

[5] Alqutub A Mozahim SF Mozahim NF Alsulami OA Alsharif SM Malebari AZ. Effectiveness and safety of intranasal corticosteroids for adenoid hypertrophy: a systematic review and meta-analysis. Int J Pediatr Otorhinolaryngol. (2025) 195(03):112450. 10.1016/j.ijporl.2025.112450 40609250

[6] Abdel-Aziz M El-Fouly M Elmagd EAA Nassar A Abdel-Wahid A. Adenoid hypertrophy causing obstructive sleep apnea in children after pharyngeal flap surgery. Eur Arch Otorhinolaryngol. (2019) 276(12):3413–7. 10.1007/s00405-019-05633-z 31520163

[7] François M. [Snoring in children]. Arch Pediatr. (2006) 13(2):207–10. 10.1016/j.arcped.2005.12.001 16406497

[8] Ferreira VHDC Miura CS Lupoli BDAC Garcia DM Lopes BCP Stelzer FG. Adenoid hypertrophy is directly associated with the severity of OSA in obese children: a pilot study. Braz J Otorhinolaryngol. (2025) 91(6):101694. 10.1016/j.bjorl.2025.101694 40753942 PMC12624784

[9] Yu Z Xu Z Fu T Liu S Cui J Zhang B. Parallel comparison of T cell and B cell subpopulations of adenoid hypertrophy and tonsil hypertrophy of children. Nat Commun. (2025) 16(1):3516. 10.1038/s41467-025-58094-w 40229254 PMC11997228

[10] Önal M Yılmaz T Bilgiç E Müftüoğlu S Sözen T Bajin MD. Possible role of apoptosis in pathogenesis of adenoid hypertrophy and chronic adenoiditis: prospective case-control study. Auris Nasus Larynx. (2015) 42(6):449–52. 10.1016/j.anl.2015.04.012 26003878

[11] Gu Z Lin Z Yang Z Zou Q Liu E Kou W. Th2-M2 polarization in the pathogenesis of adenoid hypertrophy is predominantly characterized by type 2 inflammation. Cytokine. (2026) 199:157108. 10.1016/j.cyto.2026.157108 41512631

[12] Kang J Wu Y Zhang K Yao X Wang S Xu Z. Single-cell transcriptional profiling reveals PAX5-mediated naïve B cell differentiation defect in severe adenoid hypertrophy. Exp Cell Res. (2025) 450(2):114675. 10.1016/j.yexcr.2025.114675 40675241

[13] Uçar C. [Endoscopic adenoidectomy]. Kulak Burun Bogaz Ihtis Derg. (2008) 18(2):66–8. 18628638

[14] Ripp AT Kallenberger EM Nguyen SA Schafer IV Clemmens CS White DR. Topical nasal steroids for adenoid hypertrophy in children: a systematic review and meta-analysis. Int J Pediatr Otorhinolaryngol. (2025) 198(03):112580. 10.1016/j.ijporl.2025.112580 41056645

[15] Ryazanskaya AG Yunusov AS. [Hypertrophy of adenoid vegetation in modern treatment conditions]. Vestn Otorinolaringol. (2022) 87(1):70–4. 10.17116/otorino20228701170 35274895

[16] Wang P Kong W Shan Y. The efficacy and safety of Chinese herbal compound or combined with western medicine for pediatric adenoidal hypertrophy: a protocol for systematic review and meta-analysis. Medicine (Baltimore). (2020) 99(36):e22023. 10.1097/MD.0000000000022023 32899056 PMC7478751

[17] Zhao X Xu J Wang M Hou Z Shi H Zhang X. Effect of oral Xiao-xian decoction combined with acupoint application therapy on pediatric adenoid hypertrophy: a randomized trial. Medicine (Baltimore). (2023) 102(5):e32804. 10.1097/MD.0000000000032804 36749267 PMC9901993

[18] Deng C Mo Q Zhuo X Guan Y. [Deep needling at Xiaguan (ST7) combined with electroacupuncture and warm acupuncture for adenoid hypertrophy in children: a randomized controlled trial]. Zhongguo Zhen Jiu. (2025) 45(2):179–84. 10.13703/j.0255-2930.20240630-k0002 39943759

[19] X S YG W. Systematic evaluation and meta-analysis of pediatric tuina therapy in the treatment of adenoid hypertrophy. Chinese Med Modern Dist Educ China. (2024) 22(24):101–4.

[20] KX Z G L FH WU. Meta-analysis on the treatment of adenoid hypertrophy in children with integrated traditionaChinese and western medicine. J Hainan Med Univ. (2020) 26(05):374–80. 10.13210/j.cnki.jhmu.20200207.001

[21] Y C L L D M LC L. Study on meta analysis and medication regularity of traditional Chinese medicine in treatment of adenoid hypertrophy. Chinese J Ethnomed Ethnopharm. (2023) 32(18):96–103.

[22] X X JT C Z L Y R. Meta -analysis of clinical efficacy and safety of Chinese medicine in the treatment of adenoidal hypertrophy. China Med Herald. (2020) 17(30):107–111 + 117.

[23] FJ MA XJ Y XF N. Meta analysis of the efficacy and safety of traditional Chinese medicine in the treatment of children with adenoid hypertrophy. World Latest Med Inform (Electronic Version). (2020) 18(09):96–104.

[24] Sun YL Zheng HT Tao JL Jiang MC Hu CC Li XM. Effectiveness and safety of Chinese herbal medicine for pediatric adenoid hypertrophy: a meta-analysis. Int J Pediatr Otorhinolaryngol. (2019) 119(06):79–85. 10.1016/j.ijporl.2019.01.022 30684690

[25] Liu X Jiang Z Xiao Z Jiang Y Li W Xu B. Meta-analysis of Chinese medicine in the treatment of adenoidal hypertrophy in children. Eur Arch Otorhinolaryngol. (2019) 276(1):203–8. 10.1007/s00405-018-5113-2 30361788

[26] Duan Y Li N Gu J Ma L Wu S Xie Y. Combining untargeted and targeted metabolomics to identify diagnostic biomarkers of adenoid hypertrophy. J Breath Res. (2025) 19(4):179–88. 10.1088/1752-7163/ae0dac 41027438

[27] Zhang X Li Y Wu X Lin M Wang P Zhao B. Deep learning-based automatic adenoid segmentation and a novel volume-based index for adenoid hypertrophy assessment. BMC Oral Health. (2026) 26(1):41027438. 10.1186/s12903-026-07675-2 PMC12930684 41545817

[28] Shea BJ Reeves BC Wells G Thuku M Hamel C Moran J. AMSTAR-2: a critical appraisal tool for systematic reviews that include randomised or non-randomised studies of healthcare interventions, or both. Br Med J. (2017) 358(4):j4008. 10.1136/bmj.j4008 28935701 PMC5833365

[29] Page MJ Mckenzie JE Bossuyt PM Boutron I Hoffmann TC Mulrow CD. The PRISMA 2020 statement: an updated guideline for reporting systematic reviews. Br Med J. (2021) 372(4):n71. 10.1136/bmj.n71 33782057 PMC8005924

[30] Guyatt GH Oxman AD Vist G Kunz R Brozek J Alonso-Coello P. GRADE Guidelines: 4. Rating the quality of evidence–study limitations (risk of bias). J Clin Epidemiol. (2011) 64(4):407–15. 10.1016/j.jclinepi.2010.07.017 21247734

[31] Guyatt GH Oxman AD Vist GE Kunz R Falck-Ytter Y Alonso-Coello P. GRADE: an emerging consensus on rating quality of evidence and strength of recommendations. Br Med J. (2008) 336(7650):924–6. 10.1136/bmj.39489.470347.AD 18436948 PMC2335261

[32] Balshem H Helfand M Schünemann HJ Oxman AD Kunz R Brozek J. GRADE Guidelines: 3. Rating the quality of evidence. J Clin Epidemiol. (2011) 64(4):401–6. 10.1016/j.jclinepi.2010.07.015 21208779

[33] Guyatt G Oxman AD Akl EA Kunz R Vist G Brozek J. GRADE Guidelines: 1. Introduction-GRADE evidence profiles and summary of findings tables. J Clin Epidemiol. (2011) 64(4):383–94. 10.1016/j.jclinepi.2010.04.026 21195583

[34] Granholm A Al DZ Alhazzani W Oczkowski S Belley-Cote E Møller MH. GRADE Pearls and pitfalls-part 2: clinical practice guidelines. Acta Anaesthesiol Scand. (2024) 68(5):593–600. 10.1111/aas.14384 38380849

[35] Guyatt GH Oxman AD Kunz R Brozek J Alonso-Coello P Rind D. GRADE Guidelines 6. Rating the quality of evidence–imprecision. J Clin Epidemiol. (2011) 64(12):1283–93. 10.1016/j.jclinepi.2011.01.012 21839614

[36] Nolting A Perleth M Langer G Meerpohl JJ Gartlehner G Kaminski-Hartenthaler A. [GRADE guidelines: 5. Rating the quality of evidence: publication bias]. Z Evid Fortbild Qual Gesundhwes. (2012) 106(9):670–6. 10.1016/j.zefq.2012.10.015 23200211

[37] Perleth M Langer G Meerpohl JJ Gartlehner G Kaminski-Hartenthaler A Schünemann HJ. [GRADE guidelines: 7. Rating the quality of evidence - inconsistency]. Z Evid Fortbild Qual Gesundhwes. (2012) 106(10):733–44. 10.1016/j.zefq.2012.10.018 23217727

[38] Guyatt GH Oxman AD Schünemann HJ Tugwell P Knottnerus A. GRADE Guidelines: a new series of articles in the journal of clinical epidemiology. J Clin Epidemiol. (2011) 64(4):380–2. 10.1016/j.jclinepi.2010.09.011 21185693

[39] Guo YJ Z X Z J. Systematic evaluation and meta-analysis of the safety and efficacy of massage in the treatment of adenoid hypertrophy in children. Chin Sci Tech J Data. (2022) 2(10):133–9.

[40] PL LI ZF P ZH C YN Y QJ M. The clinical effect on pediatric adenoidal hypertrophy via massage: a systematic Ｒeview. Chin J Ethnomed Ethnopharm. (2023) 32(03):109–14.

[41] Y R SX G. Meta-analysis of oral administration of traditional Chinese medicine in the treatment of adenoidal hypertrophy in children. World Latest Med Inform Digest. (2024) 24(13):110–8.

[42] Shen X Liu X Guo X Hou X Huang H Feng Z. Systematic review of Janus kinases inhibitors for rheumatoid arthritis: methodology, reporting, and quality of evidence evaluation. Front Pharmacol. (2024) 15:1459511. 10.3389/fphar.2024.1459511 39386036 PMC11461343

[43] Li L Asemota I Liu B Gomez-Valencia J Lin L Arif AW. AMSTAR-2 appraisal of systematic reviews and meta-analyses in the field of heart failure from high-impact journals. Syst Rev. (2022) 11(1):147. 10.1186/s13643-022-02029-9 35871099 PMC9308914

[44] Shea BJ Grimshaw JM Wells GA Boers M Andersson N Hamel C. Development of AMSTAR: a measurement tool to assess the methodological quality of systematic reviews. BMC Med Res Methodol. (2007) 710:15. 10.1186/1471-2288-7-10 PMC1810543 17302989

[45] Schiavenato M Chu F. PICO: what it is and what it is not. Nurse Educ Pract. (2021) 56:103194. 10.1016/j.nepr.2021.103194 34534728

[46] Yao L Sun R Chen Y Wang Q Wei D Wang X. The quality of evidence in Chinese meta-analyses needs to be improved. J Clin Epidemiol. (2016) 74:73–9. 10.1016/j.jclinepi.2016.01.003 26780259

