# Supplementary material for: Traditional Chinese medicine for pediatric adenoid hypertrophy: an Umbrella review of methodological, reporting, and evidence quality
Source: Front Allergy. 2026 May 29;7:1853582. doi: 10.3389/falgy.2026.1853582 (PMC13260284; doi:10.3389/falgy.2026.1853582)
Supplement: Supplementary file 1 [file Datasheet1.docx]

**Supplementary Table 1A** PubMed Search strategy

| Line | Search string |
| --- | --- |
| #1 | "Adenoid Hypertrophy"[Mesh] OR "adenoid hypertrophy"[Title/Abstract] OR "adenoidal hypertrophy"[Title/Abstract] OR "adenoid vegetation"[Title/Abstract] OR "adenoid enlargement"[Title/Abstract] |
| #2 | "Medicine, Chinese Traditional"[Mesh] OR "traditional Chinese medicine"[Title/Abstract] OR TCM[Title/Abstract] OR "Chinese herbal medicine"[Title/Abstract] OR "Chinese patent medicine"[Title/Abstract] OR decoction[Title/Abstract] OR acupuncture[Title/Abstract] OR Tuina[Title/Abstract] OR massage[Title/Abstract] OR "external therapy"[Title/Abstract] |
| #3 | "Systematic Review"[Publication Type] OR "Meta-Analysis"[Publication Type] OR "systematic review"[Title/Abstract] OR meta-analysis[Title/Abstract] OR metaanalysis[Title/Abstract] OR "meta analysis"[Title/Abstract] |
| #4 | #1 AND #2 AND #3 |

**Supplementary Table 1B** Web of Science Search strategy

| Line | Search string |
| --- | --- |
| #1 | TS=("adenoid hypertrophy" OR "adenoidal hypertrophy" OR "adenoid vegetation" OR "adenoid enlargement") |
| #2 | TS=("traditional Chinese medicine" OR TCM OR "Chinese herbal medicine" OR "Chinese patent medicine" OR decoction OR acupuncture OR Tuina OR massage OR "external therapy") |
| #3 | TS=("systematic review" OR "meta-analysis" OR metaanalysis OR "meta analysis") |
| #4 | #1 AND #2 AND #3 |

**Supplementary Table 2** CNKI, Wanfang, and VIP Search strategy

| Database | Search string |
| --- | --- |
| CNKI / 中国知网 | 主题=(腺样体肥大 OR 腺样体增生 OR 腺样体肥厚) AND 主题=(中医 OR 中医药 OR 中药 OR 中成药 OR 推拿 OR 按摩 OR 针灸 OR 外治 OR 中西医结合) AND 主题=(系统评价 OR Meta分析 OR 荟萃分析) |
| Wanfang / 万方 | 主题=(腺样体肥大 OR 腺样体增生 OR 腺样体肥厚) AND 主题=(中医 OR 中医药 OR 中药 OR 中成药 OR 推拿 OR 按摩 OR 针灸 OR 外治 OR 中西医结合) AND 主题=(系统评价 OR Meta分析 OR 荟萃分析) |
| VIP / 维普 | 题名或关键词=(腺样体肥大 OR 腺样体增生 OR 腺样体肥厚) AND 题名或关键词=(中医 OR 中医药 OR 中药 OR 中成药 OR 推拿 OR 按摩 OR 针灸 OR 外治 OR 中西医结合) AND 题名或关键词=(系统评价 OR Meta分析 OR 荟萃分析) |

**Supplementary Table 3** Rationale of items of AMSTAR-2

| Item | Answer | | |
| --- | --- | --- | --- |
| 1. Did the research questions and inclusion criteria for the review include the components of PICO? | Yes | No |  |
| 2.Did the report of the review contain an explicit statement that the review methods were established prior to the conduct of the review and did the report justify any significant deviations from the protocol？ | Yes | No | Partial Yes |
| 3. Did the review authors explain their selection of the study designs for inclusion in the review? | Yes | No |  |
| 4. Did the review authors use a comprehensive literature search strategy? | Yes | No | Partial Yes |
| 5. Did the review authors perform study selection in duplicate? | Yes | No |  |
| 6. Did the review authors perform data extraction in duplicate? | Yes | No |  |
| 7. Did the review authors provide a list of excluded studies and justify the exclusions? | Yes | No | Partial Yes |
| 8. Did the review authors describe the included studies in adequate detail? | Yes | No | Partial Yes |
| 9. Did the review authors use a satisfactory technique for assessing the risk of bias ^42^ in individual studies that were included in the review? | Yes | No | Partial Yes |
| 10. Did the review authors report on the sources of funding for the studies included in the review? | Yes | No |  |
| 11. If meta-analysis was performed, did the review authors use appropriate methods for statistical combination of results? | Yes | No |  |
| 12. If meta-analysis was performed, did the review authors assess the potential impact of RoB in individual studies on the results of the meta-analysis or other evidence synthesis? | Yes | No |  |
| 13. Did the review authors account for RoB in primary studies when interpreting/discussing the results of the review? | Yes | No |  |
| 14. Did the review authors provide a satisfactory explanation for, and discussion of, any heterogeneity observed in the results of the review? | Yes | No |  |
| 15. If they performed quantitative synthesis did the review authors carry out an adequate investigation of publication bias (small study bias) and discuss its likely impact on the results of the review? | Yes | No |  |
| 16. Did the review authors report any potential sources of conflict of interest, including any funding they received for conducting the review? | Yes | No |  |

Note: Based on classification scoring(Yes=1, No=0, Partial Yes=0.5)

**Supplementary Table 4** The PRISMA2020 checklists

| **Section and Topic** | **ltem** | **Checklist item** | **Answer** | | |
| --- | --- | --- | --- | --- | --- |
| **TITLE** | | | Yes | No |  |
| Title | 1 | Identity the repot as a systematic review. | Yes | No | Partial Yes |
| **ABSTRACT** | | | Yes | No |  |
| Abstract | 2 | See the PRISMA 2020 for Abstracts checlist. | Yes | No | Partial Yes |
| **INTRODUCTION** | | | Yes | No |  |
| Rationale | 3 | Describe the rationale for the review in the context of existing knowledge. | Yes | No |  |
| Objectives | 4 | Provide an explict statement of the objective(s) or question(s) the review addresses. | Yes | No | Partial Yes |
| **METHODS** | | | Yes | No | Partial Yes |
| Eligiblity criteria | 5 | Specify the inclusion and exclusion criteria for the review and how studies were grouped for the syntheses. | Yes | No | Partial Yes |
| information sources | 6 | Specily all databases, registers, websites, organisations, reference lists and other sources searched or consuted to identity studies. Specily the date when each source was last searched or consulted. | Yes | No |  |
| Search strategy | 7 | Present the full search strategies for all databases, registers and websites, incuding any fiters and limits used. | Yes | No |  |
| Selection process | 8 | Specily the methods used to decide whether a study met the inclusion criteia of the review, including how many reviewers screened each record and each report retrieved, whether they worked independently, and if applicable, details of automatin tools used in the process. | Yes | No |  |
| Data collection process | 9 | Specily the methods used to collct data from reports, including how many reviewers cllclted data from each report, whether they worked independently. any processes for obtaining or confirming data from study investigators, and if applicabie, details of automation tools used in the process. | Yes | No |  |
| Data items | 10a | List: and define all outcomes for which data were sought Specily whether all results that were compatible with each outcome domain in each study were sought (e.g. for all measures, time points, analyses), and if not, the methods used to decide which result to cllet | Yes | No |  |
|  | 10b | List and detine all other variables for which data were sought (e.g. participant and intervention characterstics, funding sources). Describe any assumptions made about any missing or unclear infomation. | Yes | No |  |
| Study risk of bias assessment | 11 | Specily the methods used to assess risk of bias in the inluded studies, incuding detais of the tol(s) used, how many reviewers assessed each study and whether they worked independenly, and if applicable, detaills of automation tools used in the process. | Yes | No |  |
| Eeffect measures | 12 | Specily for each outcome the efict measure(s) (e.g. rik ratio, mean dffrence) used in the synthesis or presentation of rsults |  |  |  |
| Synthesis methods | 13a | Describe the processes used to decide which studies were eigible for each synthesis (eg. tabulating the study intervention characerstics and comparing against the planned groups for each synthesis (item #5)). |  |  |  |
|  | 13b | Describe any methods required to prepare the data for presentation or synthesis, such as handing of missing summary satistics, or data conversions. |  |  |  |
|  | 13c | Describe any methods used to tabulate or visully display results of individual studies and syntheses. |  |  |  |
|  | 13d | Describe any methods used to synthesize result and provide a rationale for the choice(s). f meta analysis was performed, describe the model(s), method(s) to ldentit the presence and extent of statistical heterogeity and software package(s) used. |  |  |  |
|  | 13e | Describe any methods used to explore possible causes of heterogeneity among study results (e.g. subgroup analysis, meta regression). |  |  |  |
|  | 13f | Describe any sensitvity analyses conducted to assess robustness of the synthesized results. |  |  |  |
| Reporting bias assessment | 14 | Describe any methods used to assess risk of bias due to missing result in a synthesis (arising from reporting biases). |  |  |  |
| Certainty assessment | 15 | Describe any methods used to assess certainty (or confidence) in the body of evidence for an outcome. |  |  |  |
| **RESULTS** | | |  |  |  |
| Study selection | 16a | Describe the result of the search and selection process, from the number of records identlied in the search to the number of studies incuded in the review, ideall using a flow diagram. |  |  |  |
|  | 16b | Cite studies that might appear to meet the inclusion criteria, but which were excluded, and explain why they were excluded. |  |  |  |
| Study characteristics | 17 | Cite each included study and present its characteristics. |  |  |  |
| Risk of bias in studies | 18 | Present assessments of risk of bias for each included study. |  |  |  |
| Results of individual studies | 19 | For all outcomes, present, for each. study: (a) summary statistics for each group (where apropiate) and (b) an efet estimate and its precision (eg. confidence/credibile interval)l, idel using structured tables or plots. |  |  |  |
| Results of syntheses | 20a | For each synthesis, biely summarise the characteristic and risk of bias among contributing studies. |  |  |  |
|  | 20b | Present resuls of all statistical syntheses conducted. If meta -analysis was done, present for each the summary estimate and its precision (e.g. confidence/credibie interal) and measures of statistical heterogeneity. If comparing groups, describe the direcion of the efect. |  |  |  |
|  | 20c | Present results of all investigations of possible causes of heterogeneity among study results. |  |  |  |
|  | 20d | Present results of all sensitivit analyses conducted to assess the robustness of the synthesized results. |  |  |  |
| Reporting biases | 21 | Present assessments of risk of bias due to missing results (arising from reporting biases) for each synthesis assessed. |  |  |  |
| Certainty of evidence | 22 | Present assessments of certainty (or confidence) in the body of evidence for each outcome assessed. |  |  |  |
| **DISCUSSION** | | |  |  |  |
| Discussion | 23a | Provide a general interpretation of the results in the contxt of other evidence. |  |  |  |
|  | 23b | Discuss any litations of the evidence included in the review. |  |  |  |
|  | 23c | Discuss any limitations of the review processes used. |  |  |  |
|  | 23d | Discuss implictions of the results for practice, policy, and future research. |  |  |  |
| **OTHER INFORMATION** | | |  |  |  |
| Registration and protocol | 24a | Provide registration information for the review, including register name and registration number, or state that the review was not registered. |  |  |  |
|  | 24b | Indicate where the review protocol can be accessed, or state that a protocol was not prepared. |  |  |  |
|  | 24c | Describe and explain any amendments to information provided at registration or in the protocol. |  |  |  |
| Support | 25 | Describe sources of financlal or non-inancial support for the review, and the role of the funders or sponsors in the review. |  |  |  |
| Competing interests | 26 | Declare any competing interests of review authors. |  |  |  |
| Avaiabilty of data, code and other materials | 27 | Report which of the fllwing are publicy available and where they can be found: template data collection forms; data extracted frm included studies; data used for all analyses: analytic code; any other materials used in the review. |  |  |  |

Note: Based on classification scoring(Yes=1, No=0, Partial Yes=0.5)

**Supplementary Table 5** Rating of GRADE system

| Study design | Quality of evidence | Specific explanation | Lower if | Total sore |
| --- | --- | --- | --- | --- |
| Randomized trial | high | Further research is very unlucky to our confidence in the estimate of effect | Risk of bias  -1 serious  -2 very serious  Inconsistency  -1 serious  -2 very serious  Indirectness  -1 serious  -2 very serious  Imprecision  -1 serious  -2 very serious  Publication bias  -1 serious  -2 very serious | ≥0 |
|  | Medium | Further research is likely to have an important impact on our confidence in the estimate of effect and may change the estimate |  | -1 |
| Observational study | low | Further research is very likely to have an important impact on our confidence in the estimate of effect and may change the estimate |  | -2 |
|  | very low | Any estimate of effect is very uncertain |  | ≤-3 |
